# Effect of the Atmospheric Plasma Treatment Parameters on the Surface and Mechanical Properties of Carbon Fabric

**DOI:** 10.3390/ma17112547

**Published:** 2024-05-25

**Authors:** Samuele Sampino, Raffaele Ciardiello, Domenico D’Angelo, Laura Cagna, Davide Salvatore Paolino

**Affiliations:** 1 Department of Mechanical and Aerospace Engineering, Politecnico di Torino, Corso Duca degli Abruzzi 24, 10129 Turin, Italy; raffaele.ciardiello@polito.it; 2 Plasma Nano-Tech, Environmental Park, Via Livorno 60, 10144 Turin, Italy; domenico.dangelo@envipark.com; 3 Department of Science and Technological Innovation, University of Eastern Piedmont “Amedeo Avogadro”, Viale Teresa Michel 11, 15121 Alessandria, Italy; laura.cagna@uniupo.it

**Keywords:** plasma, surface treatment, carbon fiber, sustainable composites, delamination, double cantilever beam test, end-notched flexure test, flexural test

## Abstract

The use of Atmospheric Pressure Plasma Jet (APPJ) technology for surface treatment of carbon fabrics is investigated to estimate the increase in the fracture toughness of carbon-fiber composite materials. Nitrogen and a nitrogen–hydrogen gas mixture were used to size the carbon fabrics by preliminarily optimizing the process parameters. The effects of the APPJ on the carbon fabrics were investigated by using optical and chemical characterizations. Optical Emission Spectroscopy, Fourier Transform Infrared-Attenuated Total Reflection, X-ray Photoelectron Spectroscopy and micro-Raman spectroscopy were adopted to assess the effectiveness of ablation and etching effects of the treatment, in terms of grafting of new functional groups and active sites. The treated samples showed an increase in chemical groups grafted onto the surfaces, and a change in carbon structure was influential in the case of chemical interaction with epoxy groups of the epoxy resin adopted. Flexural test, Double Cantilever Beam and End-Notched Flexure tests were then carried out to characterize the composite and evaluate the fracture toughness in Mode I and Mode II, respectively. N_2_/H_2_ specimens showed significant increases in *G_IC_* and *G_IIC_*, compared to the untreated specimens, and slight increases in *P_max_* at the first crack propagation.

## 1. Introduction

Composite materials have been adopted in the automotive industry to lighten vehicles and reduce fuel consumption and therefore, to achieve a reduction in emissions [1]. The research is moving towards the use of lightweight materials that could have comparable or greater mechanical properties compared to metallic materials that are commonly adopted. The mechanical properties of composite materials depend on different factors (i.e., fiber, matrices, manufacturing processes, interface between matrix and resin and defects). The interface between the fiber and matrix in composite materials plays a crucial role in determining the overall performance and properties of the composite [2]. For this reason, surface treatments are usually used to increase the affinity of the fiber and improve the adhesion strength at the interface.

When carbon-fiber-reinforced composites are adopted as reinforcement in composite structures without any prior surface treatment, they can present low interlaminar shear strength (ILSS). This is correlated to weak adhesion and poor bonding between the fiber and the matrix.

Surface treatments are usually needed for traditional synthetic fibers, due to the fabrication processes. In carbon fibers (CFs), for example, the inertness of the surface is due to the fabrication process and its precursor. The high-temperature graphitization process causes a change in the surface morphology, with a resulting lack of functional groups on the surface, as well as excessive smoothness and fewer adsorption characteristics of carbon fibers [2].

Different solutions are used to increase the surface free energy of fibers and the adhesion to the matrix, including wet chemical methods and dry surface treatments [3].

Liou et al. [4] studied the effect of CO_2_ laser treatment with several combinations of process parameters (i.e., resolution and pixel time) on carbon-fiber fabric. Infrared spectroscopy was used to determine the increment in chemical functional groups on the fiber surface to enhance its hydrophilicity. They recorded a maximum decrement of 11.8% in contact angle. Xu et al. [5] studied two types of surface modification methods on carbon fibers: soaking and irradiating the fibers using water solution of praseodymium nitrate. The improvement in interlaminar shear strength was, respectively, 8.5 and 13.1%. Although some chemical treatments can enhance the mechanical properties of components, they usually generate chemical waste and require significant water consumption contrary to dry methods. Further, they can be less energy efficient and more prone to variability in cycle times and process control. These limitations could be overcome by using more environmentally friendly processes, such as laser or plasma treatment, which do not involve the disposal of chemical agents.

Plasma treatment has been reported as one of the most effective dry surface treatment techniques, ascribed to its ability to treat the surface retaining the bulk properties of the material, and due to its long-term durability [6].

Among various types of plasma treatment, Atmospheric Pressure Plasma Jet (APPJ) is commonly used in industrial applications for plastic, polymeric and fiber-reinforced composite surface treatment.

It is competitive in many aspects compared to similar plasma technologies, such as Dielectric Barrier Discharge (DBD). The latter operates with noble gases necessary to activate the plasma (i.e., helium or argon) while APPJ only requires technical gases, such as air or N_2_. The costs are, consequently, lower. Furthermore, the generators adopted by the APPJ systems have a low-energy impact, on the order of 1 kWh absorption, unlike those of the DBD systems which work at atmospheric pressure but with ceramic cathodes that require considerably greater power.

It is rare, nevertheless, to encounter work concerning the use of APPJ technology for the treatment of carbon fabrics rather than individual fibers. Tiwari et al. [7] used cold-remote plasma treatment on twill weave 2 × 2 carbon fabric samples, with a N_2_ and N_2_O_2_ gas mixture. As a result, polar functional groups were found, responsible for improvement in adhesion between matrix and fabric. The composite made by treated fabric and polyetherimide/polyetheretherketone/polyethersulfone (PEI/PEEK/PES) polymeric matrices showed an increase in tensile (+5.2%) and flexural modulus (+52.9%), tensile (+8.8%) and flexural strength (+16.7%), ILSS (+6.9%) and a slight decrease in tensile strength (−7%).

Jang et al. [8] used low-temperature oxygen plasma treatment to enhance the adhesion between fiber and matrix using hybrid fabrics composed of CF and PEEK fibers. The study found an increase in CF surface roughness by etching and a change in the relative content of three functional groups (hydroxyl, carbonyl and carboxyl). The adhesion strength mainly affected mechanical interlocking that yielded a 52% improvement in flexural strength and almost +17% in interlaminar shear strength at 3 min of plasma-treatment time.

Different characterization methods are usually employed to assess the effectiveness of the plasma action. Ceregatti et al. [9] applied Optical Emission Spectroscopy (OES), Raman Spectroscopy and contact angle measure for analyzing the modified fibers. In particular, the untreated carbon fabric exhibited a high contact angle with deionized water (132°), which is characteristic of hydrophobic surfaces. After the N_2_/H_2_ plasma functionalization, the wettability dramatically increased, and the most significant changes were related to the longer radio frequency plasma treatment.

Although physical and chemical characterizations provide detailed information about the modification of the surfaces, mechanical tests, such as Double Cantilever Beam (DCB), End-Notched Flexure (ENF) and Three-Point Flexural test, are generally adopted to evaluate the effective increase in the mechanical properties of the materials. They provide indications about the maximum values of tension at the failure or energy release rate of the composites. The procedure of the DCB and ENF tests is standardized and presented in ASTM D5528 [10] and ASTM D7905 [11], respectively. The interlaminar fracture toughness is characterized by the critical strain energy release rate for Mode I and II, *G_IC_* and *G_IIC_*.

The increase in the G_c_ parameters following APPJ plasma treatment leads to an increase in resistance to delamination with related improvement in component structural integrity and durability: improved adhesion between composite constituents has been verified first by chemical surface characterization and subsequently by mechanical functionalization testing.

Novotná et al. [12] explored the potential of plasma treatment to enhance the wettability of recycled carbon fibers to allow the covalent bond formation between them and epoxy resin. Mechanical properties changes (impact toughness and three-point bending test) of CF/epoxy samples were analyzed. It was found that the modulus of elasticity increased (+21–29%), as well as bending stress (+26–31%), thanks to the surface treatment and the increasing content of recycled carbon fibers while an increase in the surface roughness of the fibers and the oxidation of their surface was defined through surface characterization method.

Zaldivar et al. [13] explored the potential of atmospheric plasma treatment to enhance adhesively bonded composite joints, revealing its promise as an alternative to current surface preparation methods. The study investigated the sensitivity and optimization of plasma processing parameters using a graphite–epoxy composite. Findings outlined that plasma-treated specimens exhibited superior bond strength and fracture resistance *G_IC_* compared to conventionally prepared specimens, starting from 280 J/m^2^ to 525 J/m^2^. 

The present paper aims to contribute to the development of the APPJ treatment on carbon fabrics to enhance the mechanical properties of CF/epoxy composites in terms of increased wettability of CFs and adhesion with the bio-epoxy matrix adopted.

To the authors’ best knowledge, this work is the first to study the effect of the APPJ on twill fabric and not on the single fiber to increase the mechanical properties of composite materials. Although treating the single fiber is effective on the mechanical properties, practically it is not a viable solution because the effect of the treatment abruptly decays [14]. The effect of the APPJ treatments and their optimal calibration are studied and critically analyzed from a chemical and mechanical point of view by means of spectroscopic analysis, DCB, ENF and flexural testing. The difference between the untreated and treated composites is finally highlighted.

## 2. Materials and Methods

### 2.1. Materials

Composite specimens were manufactured by resin vacuum infusion starting from commercial 210 g ProFinish 3k carbon fiber 2 × 2 twill fabric (Easy Composites, Stoke on Trent, UK) [15]. The resin is an epoxy resin with 31% bio-content IB2 (Easy Composite, Stoke on Trent, UK). The bio-content of the resin is due to the glycerol, plant-derived in place of petroleum-based propylene, and it presents the mechanical properties reported in Table 1. The reverse side presents a resin binder applied by the producer to ensure weave alignment during handling and mold positioning. Further, the binder is used by the manufacturer to facilitate the cutting and joining, common in prepreg parts.

**Figure 1:**
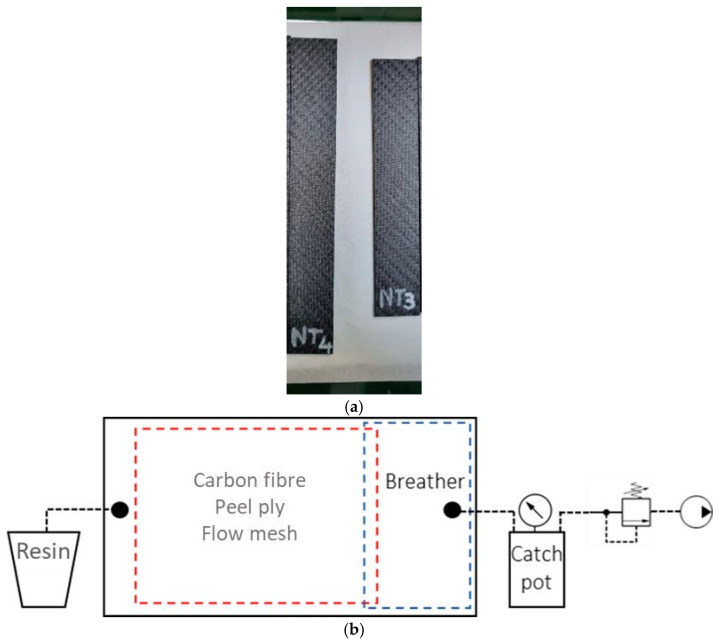
(**a**) ENF (left) and DCB (right) CFRP specimen fabricated by resin vacuum infusion process, made by untreated (NT) fabrics; (**b**) schematic diagram of the resin vacuum infusion process.

The laminate plates were produced 12 h after plasma treatment of the fabrics (by storing the treated fibers in a vacuum bag). They were made at the laboratory of the Department of Mechanical and Aerospace Engineering (DIMEAS) of the Polytechnic of Turin. Twelve layers for each of the three laminates were placed on a glass plate mold. Three different batches were prepared by using fabrics plasma treated with N_2_, fabrics treated with N_2_/H_2_ and without any treatment. The layers were arranged keeping the same orientation. Sealant tapes were placed to secure the vacuum bag, a peel ply layer was used to release the laminates after curing and a flow mesh layer was used to ensure the uniform flow of the resin through the fibers. A schematic diagram of the infusion process, as well as some fabricated specimens, are shown in Figure 1.

For DCB and ENF specimens, a folded layer of aluminum 9.8 μm thick was placed between the sixth and seventh layers to prepare the crack. Wax was applied on each surface of the aluminum sheet to avoid bonding during the infusion process and to leave the cracked end part of the sample open, as requested by the ASTM standard.

The DCB sample dimensions were 140 × 25 × 2.8 mm, and the cracked end part was 50 mm long.

The sample dimensions of ENF were 170 × 25 × 2.8 mm, and the cracked end part was 65 mm long. At least three specimens were tested for each laminate.

Specimens fabricated for the Three-Point Flexural test were 80 × 12 × 2.8 mm without any cracked parts.

### 2.2. Experimental Test

#### 2.2.1. Atmospheric Pressure Plasma Jet Treatment

Plasma treatment was performed with an OpenaAir^®^ plasma jet system PFW1004 of Plasmatreat GmbH (Steinhagen, Germany). The generator was the FG5001, which generated constant tension with a supply voltage of 100–240 V, equipped with modern insulated-gate bipolar transistor semiconductor final stages combining high performance with precise plasma power regulation and control functions. One hundred percent of the reference voltage was used with resultant minimum electrical pulsation, assuring a stable discharge at 25 kHz–15 A. Plasma Jet system mounted the rotative nozzle RD1004 (Plasmatreat GmbH, Steinhagen, Germany) series to generate the cold plasma. The pressure of the ejected gas was 4 bar. A 3-axis movement system, Janome JR3000 (Janome Industrial Equipment, Tokyo, Japan) series, was used for the plasma jet motion with a proper path set by the user. It was designed so that a single layer of fabric (35 × 35 cm) could be processed in just under 4 min, under conditions that would not damage the fabric itself.

To ensure proper control over the activation of the treated fabrics, it is essential to minimize the time elapsed between the activation process and the fabrication through vacuum resin infusion. This was conducted to prevent uncontrolled interaction of the activated fabric functionalization with the surrounding air. Consequently, the sheet production process was carried out after 12 h.

Gas choice and power specification are ascribed as the primary plasma parameters. The voltage of the machine (plasma voltage [V] in Table 2) is indirectly controlled by acting on the voltage percentage (reference voltage [%] in Table 2) to be used; this variable, in conjunction with the management of the plasma excitation frequency (plasma intensity/frequency [kHz] in Table 2), makes it possible to obtain an ionization power of the plasma compatible with the result pursued. Distance jet sample, jet speed and gas flow are the secondary parameters that were chosen. Both primary and secondary parameter controls ensure maximum process efficiency by regulating discharge species concentration and intercepting primary fire zones with higher densities of ionized species.

The N_2_/H_2_-based plasma (5% of hydrogen content) was proven to be more exothermic, so it was deemed necessary to increase the distance from the sample to be treated to maintain its structural integrity.

#### 2.2.2. Optical Emission Spectroscopy

Optical Emission Spectroscopy (OES) is a key technique for analyzing the APPJs’ chemical composition by measuring the optical emissions from excited species within the plasma. In N_2_ and N_2_/H_2_-based APPJs, OES is essential for studying plasma kinetics and chemistry, aiding in the optimization of plasma parameters for various applications. A spectrometer Ocean Optics LIBS2500 Plus (Ocean Optics, Inc., Dunedin, FL, USA), fiber optic cable and OceanView computer software (OceanView 2.0.8 version) were used to examine species spectra in the sensitive range from 150 to 1100 nm as wavelength.

The OES was performed before investigating the effects of plasma on the surface of the carbon fabrics. The probe was placed in proximity to the plasma jet such that it operated in an overpressure environment condition. This condition provides an indication of the capability of the plasma flow without any influences by the air surrounding the treatment chamber: the OES shows the potential ionizing effect of the chemical species involved and their variation with the primary plasma parameters. This provides preliminary guidance on the choice of parameters to be adopted in combination with preliminary experiments.

#### 2.2.3. Fourier Transform Infrared-Attenuated Total Reflection and Raman Spectroscopy

Fourier Transform Infrared-Attenuated Total Reflection (FTIR-ATR) spectroscopy equipment was adopted as a characterization method to investigate the presence of chemical structures (i.e., functional groups, active sites) on the surface of the sample before and after treatment. The machine used was an IRSpirit model equipped with QATR-S (single-reflection ATR accessory) provided by Shimadzu GmbH (Kyoto, Japan).

Micro Raman–LabRam HR Evolution machine (Horiba Jobin Yvon, Paris, France) acquired information about possible surface organic and inorganic compounds on the sample surface, and the structural changes in the material by exploiting the polarization variations in the molecular bonds. It is equipped with a confocal microscope and a charge-coupled device detector.

The spectra were acquired with a neodymium-doped yttrium aluminum garnet (Nd:YAG) green laser source (wavelength 532 nm). A micro-optical zoom 50–80× was mounted. Stokes and anti-Stokes spectral features could be simultaneously measured, providing additional information. The spectral range was between 1700 and 100 cm^−1^. This choice is justified by the fact that it was desired to optimize the spectral resolution by clearly discriminating neighboring spectral peaks, and also to concentrate the molecular information of interest for this type of material, centered in this frequency range.

Peak intensities of G (order) and D1 (disorder) graphitic bands were calculated, as well as peaks deconvolution.

#### 2.2.4. X-ray Photoelectron Spectroscopy Characterization

Surface chemical modifications induced by APPJ treatments were determined by XPS analysis. 

XPS spectra were recorded with a Versa Probe I Physical Electronics (ULVAC-PHY, Chanhassen, MN, USA) instrument, equipped with an aluminum target, monochromatic Al Kα radiation, hυ = 1486.6 eV. The core lines (C1s, O1s) were acquired at 150 eV pass energy, which leads to an energy resolution of 0.4 eV. After a linear-type background subtraction, the spectra were fitted using a non-linear least-squares fitting program adopting a Gaussian–Lorentzian peak shape. C1s and O1s spectrum peaks were subsequently examined by peaks deconvolution.

The untreated, N_2_-based plasma-treated and N_2_/H_2_ mixture plasma-treated samples (1 cm^2^) were analyzed. 

#### 2.2.5. Digital Image Correlation Preparation, Setup and Crack Monitoring Code

One aspect that remains challenging is the automatic crack tip monitoring during propagation when the test is in progress. The Digital Image Correlation (DIC) allows the monitoring of the crack growth for Mode I and II delamination tests and consequently makes it possible to have a more immediate evaluation of the fracture toughness vs. crack length value.

DIC is a non-contact optical technique that allows for obtaining information about deformation and displacement fields within a region of interest (ROI). VIC 3D (Correlated Solution, Irmo, SC, USA) version 6.2. software was used for the stereo-DIC elaborations.

During the deformation process, two high-resolution cameras acquire images at a high acquisition frequency rate. The images are then processed by image correlation between the n − 1 and n consecutive images in terms of grey intensity change. This provides an analysis of the ROI and the resulting changes in the range of displacements and deformations [17,18].

Specimen images from the DCB test were processed DIC. Initial calibration, ROI selection and subset subdivision were necessary for successful correlation. Initial calibration, region of interest (ROI) selection and subset subdivision were necessary for successful correlation. A first guess point was required to aid the correlation process under high-strain conditions. 

**Figure 2:**
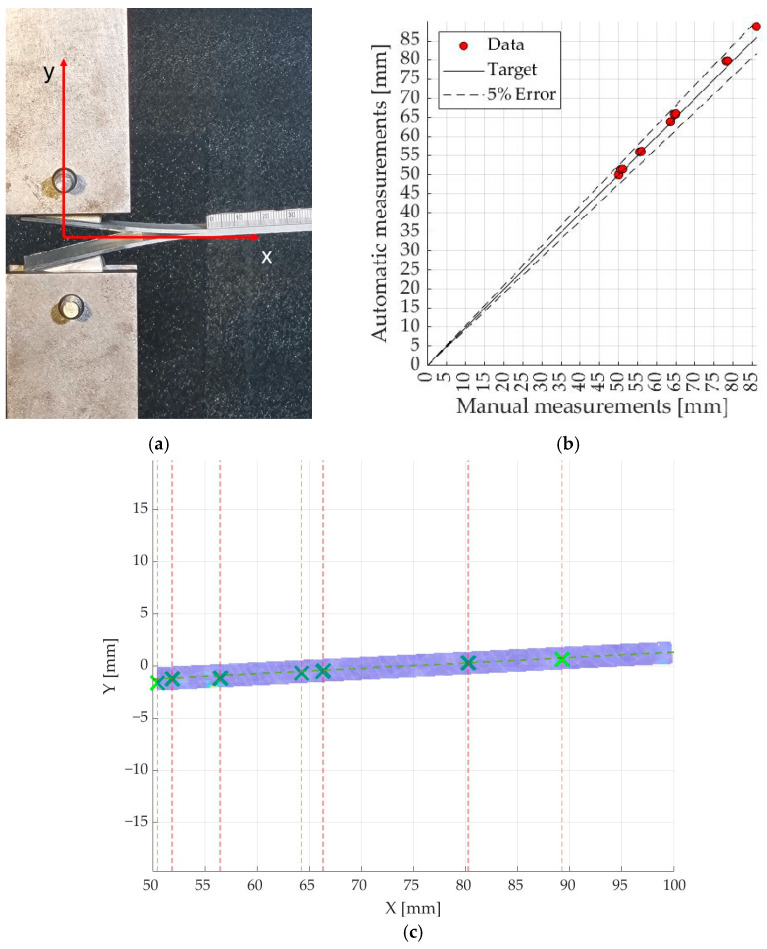
(**a**) DCB test specimen with xy coordinates exploited by DIC analysis; (**b**) automatic vs. manual crack tip measurement; (**c**) crack tip location (green crosses) in ROI.

Aluminum loading blocks were used to load the specimens, as shown in Figure 2a. The displacement outputs and the deformation ε_yy_ were required for the automatic identification of different positions of the crack tip and its progression using Matlab^®^ R2021b code. The code searched among the X,Y coordinates of each subset where the deformation ε_yy_ was maximum and excluded false positives. A position selector was inserted in the ROI close to its longitudinal axis to save the coordinates of the positions in a specific vector. The outputs were compared with the positions of the crack tip observed manually using millimeter scales on the specimen. For one of the specimens fabricated with untreated carbon fabrics, the method was considered acceptable as the differences between the two measurements did not exceed 5% of variability as shown in Figure 2b. The ROI blue area and successive positions of the crack tip are shown in Figure 2c. 

#### 2.2.6. Fracture Toughness

A Mode I delamination DCB test was performed with an MTS loading machine with a 500 N load cell installed on the upper mobile crossbar with a loading speed of 5 mm/min. The test was carried out according to the ASTM D5528 [10] standard by means of the Compliance Calibration (CC) method. DIC worked in parallel with a frame rate of 1 Hz. 

For the Mode II delamination 3-point ENF test, a Zwick/Roell Z005 machine (Zwick Roell Group, Ulm, Germany) was used with 5 kN load cell mounted. Test control and data acquisition were performed using TestXpertII 3.2 software. Two different steps (non-precracked NPC and precracked PC) of loading and unloading phases were recorded for each specimen, according to the ASTM D7905 and CC methods. A speed of 0.5 mm/min was set.

The resistance of the material to delamination is determined by critical strain energy release rate or interlaminar fracture toughness G_C_ computation: *G_IC_* for Mode I and *G_IIC_* for Mode II.

In particular, Mode I interlaminar fracture toughness is computed as follows:

(1) $$G_{I}=\frac{mP\delta \;}{2ba}\frac{F}{N}$$

where *P* is the load, *δ* is the displacement related to it at each crack length registered, *b* is the specimen width, *a* is the crack length, *m* is the slope of the load–displacement curve and *F* is the parameter that corrects the effects of large displacement at fracture while *N* a parameter that accounts for large displacements and fracture and for stiffening of the specimen by the load blocks or piano hinges. The latter was chosen as a fixture for the load transmission to the specimen.

Mode II interlaminar fracture toughness was computed as follows:

(2) $$G_{II}=\frac{{3mP}_{max}^{2}}{2b}a^{2}$$

where *P_max_* is the maximum load computed at the first propagation of a for the NPC Fracture test and the second propagation for the PC Fracture test, according to the ASTM standard, *m* is the slope of the load–displacement curve evaluated from the preliminary CC phase obtained thanks to linear least squares regression analysis and *b* is the width of the specimen.

Specifically, the *C*-*a*^3^ diagram is obtained using the first compliance calibration loading phases as outlined in ASTM D7905 [11] standard; the values of angular coefficient *m* and intercept *A* are needed for the calculation of *G_IIC_* for NPC and PC test and are obtained by considering the compliance C, which is the inverse of the angular coefficient of the loading curve *P*–*δ* in the first elastic portion, and a corresponds to the crack length for the 3 different test specimen placements with respect to the support used for the ENF test (*a*_0_ = 20–30–40 mm).

This time the crack length propagation was observed with a high-resolution camera.

*G_IIC_* for NPC and PC fracture tests are determined by first calculating candidate toughness *G_Q_*, equally evaluated according to Equation (2), and *G_IIC_* = *G_Q_* if %*G_Q_* turns out to be 15 ≤ %*G_Q_*_(NPC/PC)_ ≤ 35, as described in the standard. Candidate toughness %*G_Q_* is determined using

(3) $${\%G}_{Q,j}=\left[\frac{100{\left(P_{j}a_{j}\right)}^{2}}{{\left(P_{max}a_{0}\right)}^{2}}\right];\;\;j=1,2$$

where *%G_Q,j_* are the two values of *G_Q_* associated with the two compliance tests, *P_max_* is the max load taken from the fracture test, *P_j_* is the peak value of the force achieved during CC at *a_j_*, and *a_j_* is the *j*th crack length used during CC.

#### 2.2.7. Flexural Test

Flexural tests according to ASTM D790 [19] standard were conducted on carbon-fiber-reinforced composite specimens to evaluate the potential effect of plasma treatment by using traditional standard tests. 

Again, the tests were conducted on composite materials prepared with untreated, nitrogen-treated, and nitrogen-hydrogen-treated fibers.

Testing was performed using a Zwick-Roell Z005 test machine (Zwick Roell Group, Ulm, Germany) equipped with a 5 kN load cell. Test control and data acquisition were performed using TestXpertII 3.2. software.

The support length *L* was determined based on the specimen thickness *t*, following ASTM specifications, as 16 times the thickness. 

## 3. Results and Discussion

OES was performed on the plasma jet source while FTIR-ATR and Raman Spectroscopy were carried out on untreated and plasma-treated fabric samples for chemical and physical characterization. The presence of functional groups and chemical species on the surface was investigated. Once the characterization results were analyzed, the process parameter setup was confirmed, and the fabrics were then treated with the two configurations presented in Table 2. The samples needed for mechanical characterization were thus fabricated.

### 3.1. Surface Chemical Characterization

#### 3.1.1. Optical Emission Spectroscopy

Emissivity analysis was conducted as a function of the ionization power of the gas carrier used to generate the plasma at a specific gas flow rate of 31 L/min. The analysis covers a specific range of operating powers controlled by the frequency set on the machine, 23–25 kHz.

The absence of oxygen peaks is due to the position of the probe relative to the plasma jet, in an overpressure condition. Traces of oxygen were revealed by surface spectroscopies that consider the plasma with the treated fabric.

The OES scan revealed three distinct spectral regions and peaks within the plasma, corresponding to N_2_ second positive system/NH, Hα and N_2_ first positive system. In the spectral range of 300–400 nm, several peaks were observed and attributed to the second positive system of molecular nitrogen (C^3^∏^+^u-B^3^∏^+^g) [20], with the most intense peak occurring at 336 nm corresponding to the NH*, different from the N_2_ typical peak of N_2_ plasma source at 337 nm [21]. This peak could be associated with specific electronic and molecular transitions of NH, NH_2_ or NH_3_ excited in plasma.

**Figure 3:**
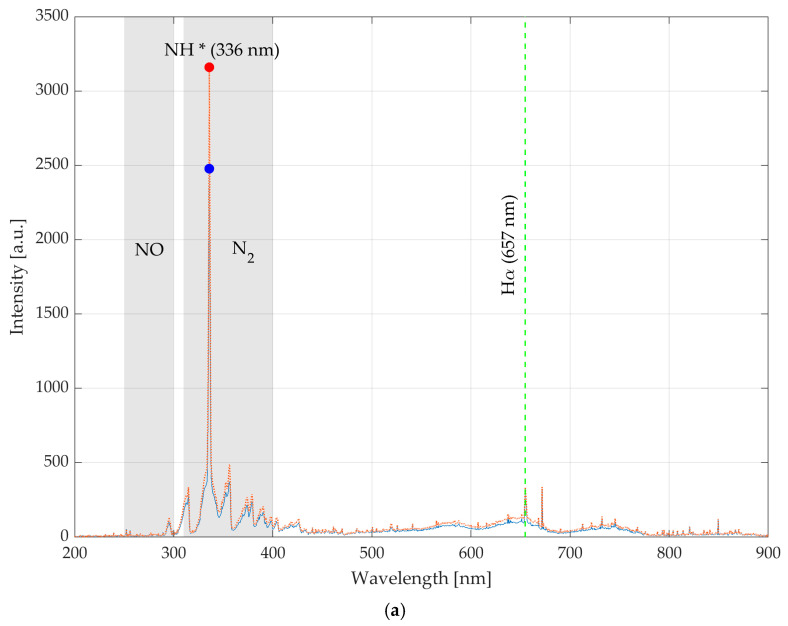

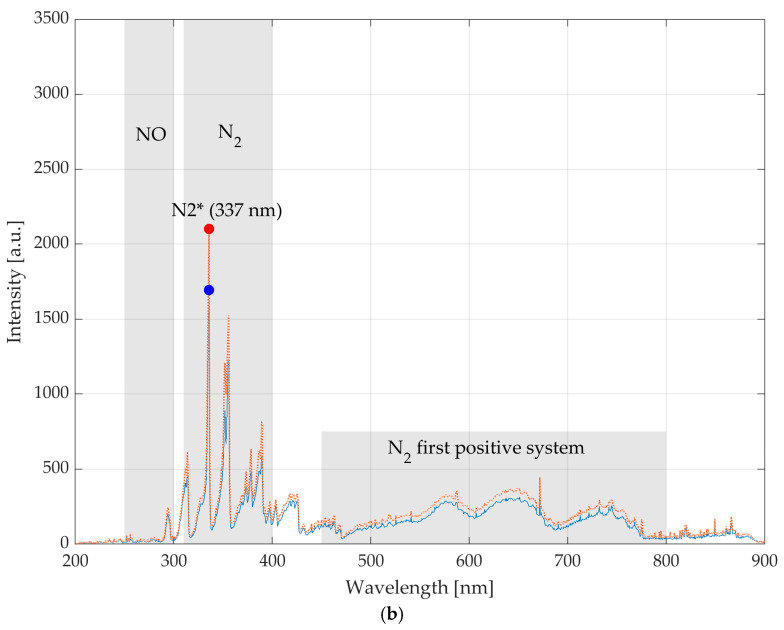
(**a**) OES spectra for N_2_/H_2_ plasma ionization gas (*Q* = 31 L/min) at *f* = 23 kHz (blue) and *f* = 25 kHz (red); (**b**) OES spectra for N_2_ plasma ionization gas (*Q* = 31 L/min) at *f* = 23 kHz (blue) and *f* = 25 kHz (red).

Figure 3a shows the intensity value of specific chemical groups emitted by the N_2_/H_2_ gas and recorded by the spectroscopic probe in the case of *f* = 23 kHz and *f* = 25 kHz at a fixed gas flow rate of 31 L/min. 

The peaks related to nitrogen traces (N_2_ second positive system 315, 357 and 375 nm), NH* (336 nm) and hydrogen-based Hα (657 nm) are highlighted since they are fundamental elements for adhesion with the epoxy groups typical of the epoxy resin used for the final laminate fabrication. In particular, the intensity value of NH* with operative *f* = 25 kHz is +27% related to the case with operative *f* = 23 kHz. The NH* peaks are highlighted in Figure 3a with red and blue points respectively for the *f* = 25 kHz and *f* = 23 kHz conditions. Identification of Hα trace is facilitated with a green dashed line.

Figure 3b, on the other hand, displays the spectrum obtained through OES analysis of the plasma source N_2_ in the same operative condition of *f* = 23–25 kHz and 31 L/min of gas flow rate. Here, the trace of the second positive system is more pronounced, especially the peak at 337 nm of N_2_* that covers the NH* peak at 336 nm. A decrease in gas flux, within a certain range, improves ionization in the plasma: with less gas, there is a greater probability that gas particles will interact with each other than with the plasma energy, resulting in greater ionization and thus an increase in the intensity of spectral peaks.

The spectral region between 450 and 800 nm is characterized by accentuated spectral peaks. These peaks could be attributed to the N_2_ first positive system [22].

The intensity of N_2_* = 337 nm of *f* = 25 kHz case is increased by 24% with respect to the case of *f* = 23 kHz. The N_2_* peaks are highlighted in Figure 3b with red and blue points respectively for the *f* = 25 kHz and *f* = 23 kHz conditions. 

These results have provided crucial insights for the choice of the process parameters to be adopted.

#### 3.1.2. Fourier Transform Infrared-Attenuated Total Reflection and Raman Spectroscopy

**Figure 4:**
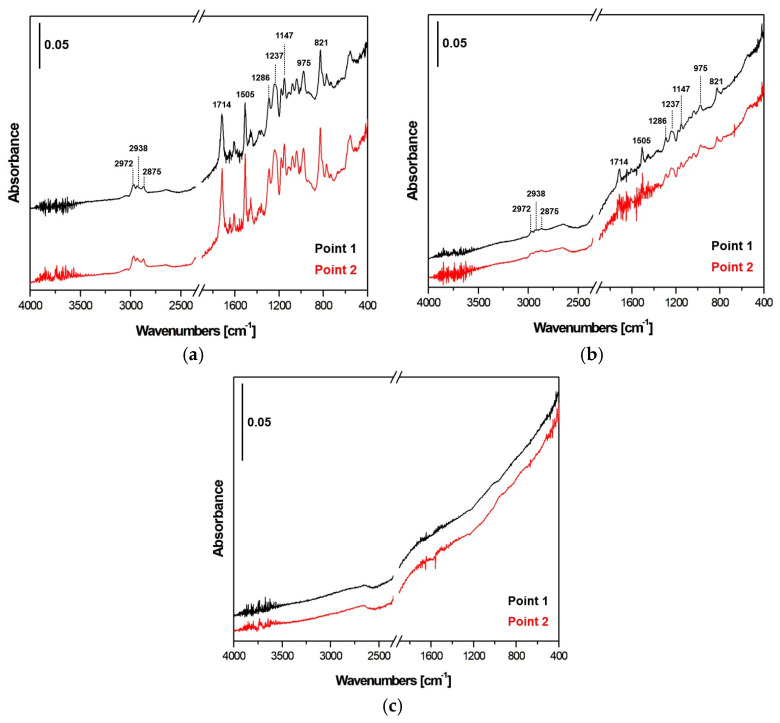
FTIR-ATR spectra: (**a**) untreated fabric, (**b**) N_2_ plasma treated and (**c**) N_2_/H_2_ plasma treated fabrics.

FTIR-ATR was conducted on the fabric sample surfaces at two different points of observation, equidistant from the center to assess the effectiveness of the treatment along the surface and to evaluate possible local effects. Slight differences among the three types of the investigated samples were observed. Figure 4a–c shows the spectra of the FTIR-ATR analysis for the untreated, N_2_ and N_2_/H_2_ treated samples, respectively. A marker is reported to quantify the amplitude of the recorded peaks and make appropriate evaluations by comparing the spectra of different samples.

The spectra illustrate particular differences between treated and untreated samples. The peaks detected between 2875 and 2972 cm^−1^, typical of CH_2_ and CH_3_ bonds, are ascribed to an unsaturated polyester, poly(4,4-dipropoxy-2,2-diphenylpropane fumarate) since high compatibility of the chemical composition has been found in the software database, LabSolutions IR [23]. 

It is related to a base pretreatment, commonly adopted in fiber composite, to allow the handling of the fibers and avoid their distortion. Further, it has been found that this pretreatment slightly increases the interlaminar fracture toughness, but it is detrimental to the mechanical resistance of the final composite: an incorrect amount of binder (i.e., amount by weight, %w) can cause it to leak beyond the tows region and thus blocks the flow of resin, hence diminishing the permeability of the fiber preform resulting detrimental to the manufacturing process. In particular, it can affect the distribution of properties, such as stiffness, resulting in structural inhomogeneity [24]. These peaks are decreased after plasma N_2_ treatment and entirely removed after N_2_/H_2_ treatment: in the former case, the decrease is due to the oxidative action of surface organics, while in the latter case, the total removal of these trace organics is most likely due to a surface pyrolysis reaction.

In addition, peaks associated with molecular vibrations of carbon bonds are reported in the shorter wavelength zone (<1800 cm^−1^): 1714 cm-1 for C=O, 1505 cm^−1^ for aromatic C=C bonds, 1286–1147 cm^−1^ for C-N, 1237 cm^−1^ for C-O, 975–821 cm^−1^ for C-H and simple C-C bonds. 

The oxygen of the surrounding air reacted with carbon: free radicals, which had not yet decayed, acted by binding to oxygen in the air since a certain distance was set between the plasma jet and fabric. This result was also noted by Ciardiello et al. [25] who showed that polypropylene surfaces treated with nitrogen-based plasma exhibited a high content of oxygen that was related to the reaction with the air in the environment of the plasma treatment. Nonetheless, the induced polar functional groups and radicals formed onto the surface after combination with oxygen allow the wettability of strongly nonpolar carbon fabrics to be increased by working in parallel with the direct functionalization achieved with the nitrogen-based gas. The traces of carboxyl terminus groups detected are characteristic in the case of chemical interaction with nitrogenous groups of the epoxy resin: covalent bond formation through esterification reactions, polarity and surface energy enhancement leads to better wettability. Traces of single bonds are found in the vicinity of 1290 cm^−1^.

Moreover, a change in the group C=C close to 1650 cm^−1^ is visible in the treated fabrics. This latter seems to have undergone different variations for the two treatments. In the N2 case, ablation is predominantly acting on the outer low-binding energy structure characterized by the lack of active carbon elements. In the N_2_/H_2_ case, plasma acted most directly and penetratingly on the internal structure of CFs, known for its binding symmetrical carbon elements. The hydrogen contribution could promote deeper interactions and specific chemical reactions within the material, affecting the core structure [26]. 

#### 3.1.3. Micro-Raman

Changes in the graphitic structure of the samples were investigated using Micro-Raman spectroscopy through the carbon order and disorder bands evaluation. The intensity ratio *R* was studied as the ratio between the disorder D1 band typically placed at 1330–1360 cm^−1^ and the order G band at 1550–1590 cm^−1^. The *R* factor provides information about the crystalline structure and order of the material. The D1 band is associated with structural disorder and the presence of defects in the material. The G band is related to regions of crystalline order in the carbonaceous material. The change in *R* can be related to changes in the reactivity of the carbonaceous material: increase in reactivity and defects, changes in surface wettability, and relation to functional groups.

**Figure 5:**
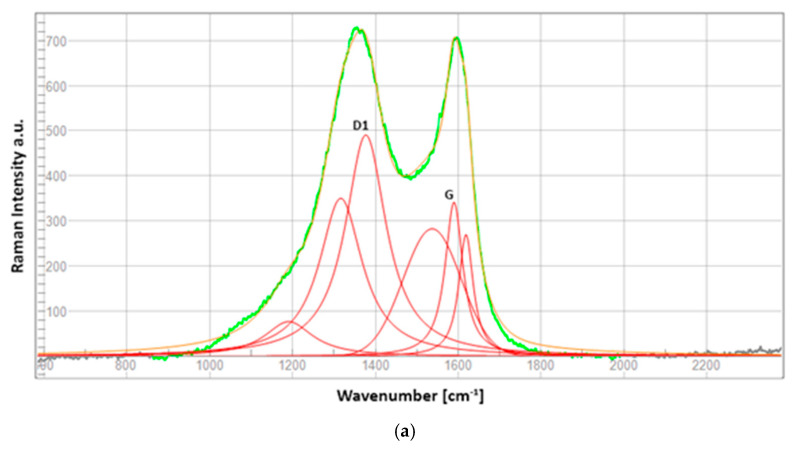

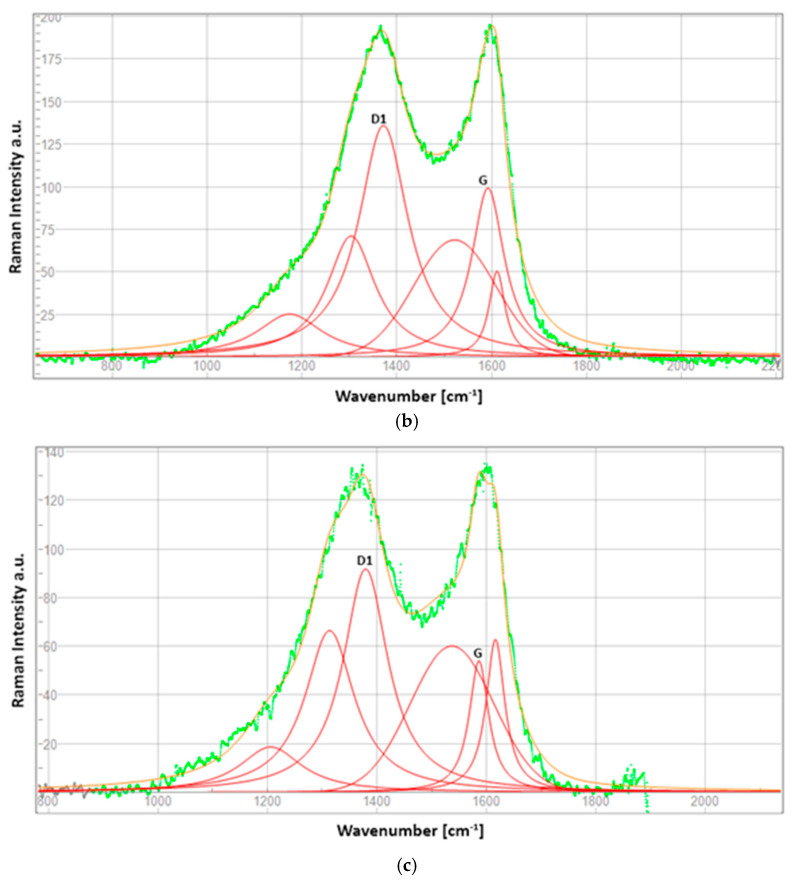
Raman spectra deconvolution of G and D1 bands in 700-2200 cm^−1^ wavenumber window: (**a**) untreated, (**b**) N_2_ treated and (**c**) N_2_/H_2_ sample.

The deconvolution curves of the D1 and G bands were investigated, as shown in Figure 5a–c, through Raman intensity–wavenumbers diagrams. The yellow-colored curve represents the deconvolution of the green curve while the red peaks depict the molecular composition of the sample surface and they are specific to the primary order band G and primary disorder band D1, whose ratio defines *R*; the other red curves are characteristic of the secondary disorder bands.

These curves are average curves measured at four different points on the front side of the samples, and the values recorded were *R_NT_* = 1.43, *R_N_*_2_ = 1.37 and *R_N_*_2/*H*2_ = 1.55. Further information could be obtained by looking at the Raman band shifts, listed in Table 3: the peak position upshifts to higher frequencies are typical of bonds subjected to tensile stress. In contrast, the downshift of peaks is related to the compressive stress on the chemical bonds that react with certain shortenings [27].

The lowest *R* result obtained for the N_2_ case indicates that the material has not been massively affected by the surface treatment, maintaining a certain degree of order. A further explanation is related to the cleaning of the resin binder (organic molecule fragments), for which its removal has allowed the fibers on the outermost layer [28]: it reverts to the graphitic-amorphous type characterized by high levels of disorder and is thus more reactive, unlike the more hydrophobic crystalline graphite.

The increase in *R*-factor and downshift of G-band peaks then leads to the formation of new crystal structures as a result of the surface chemical-morphological change, thus allowing to increase in the characteristics of adhesion with the resin through mechanical interlocking and wettability [29].

As previously anticipated, the N_2_/H_2_ plasma treatment seems to have acted directly on the core of the structure, increasing the level of disorder in the structure between the molecular planes: the ratio *R* is the greatest, confirming the downshift of the peak of G. The increase in I_D_/I_G_ is assigned to a modification of the balance in the sp^2^ and sp^3^ C atoms on the surface due to the plasma functionalization [30], and it is highlighted in the outcomes of the XPS analysis.

#### 3.1.4. X-ray Photoelectron Spectroscopy 

Surface chemical modifications obtained through plasma treatment were also characterized by XPS analyses. Table 4 shows the O and C percentages, as well as O/C ratios, obtained from the quantification of XPS core peak areas for untreated CF fabric samples and treated samples after N_2_ and N_2_/H_2_ treatments under different processing conditions. In the first row, the C and O atomic percentages and the O/C ratio of the untreated sample are reported for reference. 

**Figure 6:**
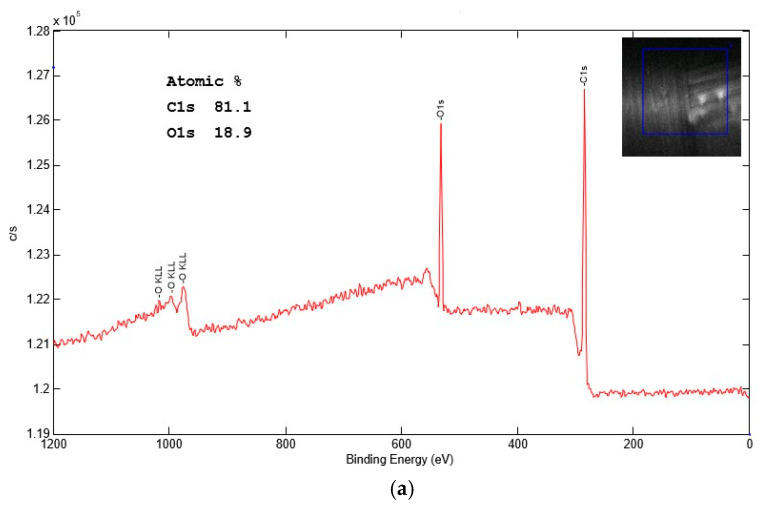

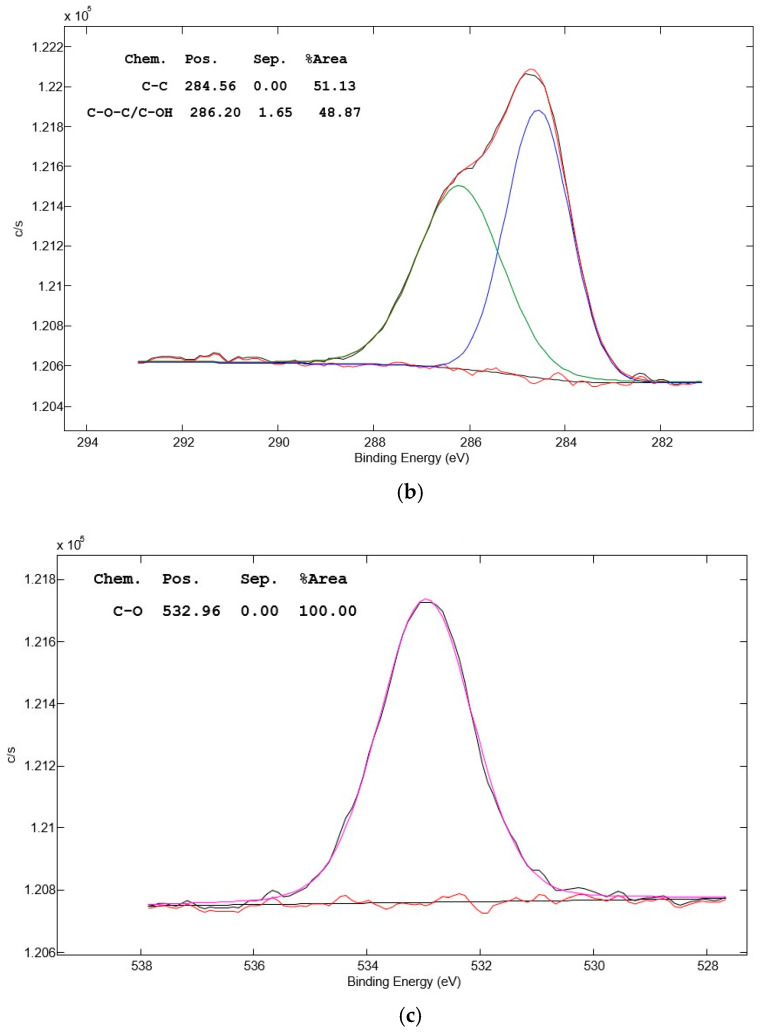
XPS spectra of untreated sample: (**a**), C1s (**b**) and O1s (**c**) core lines deconvolution.

The XPS core lines of C1s and O1s were studied in detail for all the samples. For the untreated fabric, the deconvolutions of these traces are displayed in Figure 6, while all the results are listed in Table 5, with the assignment of the different components in terms of chemical states. The XPS spectra present the strength of the electronic signal on the y axis and electron binding energy on the x axis. 

In particular, Table 4 displays that the O/C level after N_2_ treatment increased (from 0.23 to 0.35) while it decreased after N_2_/H_2_ treatment with respect to the untreated sample (from 0.23 to 0.14). 

In the former case (N_2_ treatment) there is evidence of an oxidizing effect, probably related to the recombination of metastable nitrogen with oxygen from the air (afterglow region) [31]. In contrast, the plasma generated with the N_2_/H_2_ mixture presents a reduction in surface area, with a consequent reduction in oxygen content.

In support of this claim, deconvolution of the C1s peak in the N_2_ plasma treatment features a different repartition of carbon-oxygen species highlighting, in particular, the presence of functional groups C=O (7.55%, 288.23 eV) and COOH (4.32%, 289.77 eV) absent in the as-received samples. 

Similarly, deconvolution of the O1s peak of the treated sample evidences peaks of the functional groups C=O (32.24%, 531.38 eV) and COOH (11.97%, 533.74 eV), as shown in Table 5.

The deconvolution of C1s and O1s peaks in the N_2_/H_2_ plasma treatment, despite a sharp decrease in oxygen content on the surface, presents a similar species distribution as before.

These elements concur, together with what is shown in the FTIR-ATR analysis, to evidence the fragmentation of the surface binder, which in one case occurs in a strongly reducing environment (N_2_/H_2_) while in the other partially oxidizing (N_2_) due to atmospheric oxygen in the afterglow zone.

The presence of hydrogen in the N_2_/H_2_ mixture turns this type of plasma extremely more aggressive toward the fibers leading to a deeper structural modification evidenced by a percentage of π-π bonds [32] of 8.01% compared to 0.79% of the same in the N_2_ case.

### 3.2. Mechanical Characterization

#### 3.2.1. Fracture Toughness *G_IC_*–Double Cantilever Beam Test

**Figure 7:**
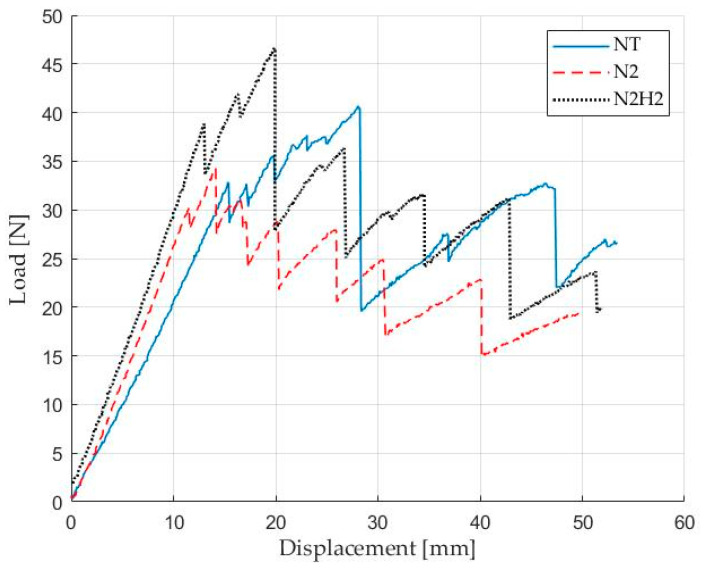
DCB test–Load vs. displacement for untreated NT sample (blue), N_2_ (red) and N_2_/H_2_ (black) treated sample.

Representative load–displacement plots (Figure 7) showed different crack propagations depending on the treatment of the specimens with N_2_ showing the lowest instability during delamination. 

**Figure 8:**
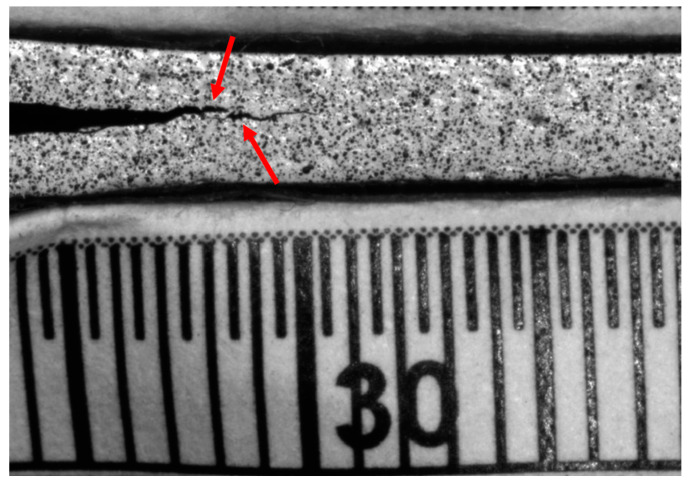
Formation of secondary crack (right side red arrow) near the main crack tip (left side red arrow).

Stick-slip behavior [33], which is the temporary stops and resumptions of crack propagation and the formation of secondary cracks, was observed for the samples fabricated with NT fabrics as clearly visible in Figure 8: the complex fracture behavior of carbon-fiber-reinforced composite materials resulted in NT samples with non-unique crack fronts and is further confirmed by the trend of the load–displacement curve in Figure 7. Furthermore, the numerous load drops before the maximum load is reached indicate the presence of secondary cracks forming during the propagation of the main crack. On the other hand, the higher frequency and moderate amplitude of load drops in the case of the other two families of specimens is related to the growth of the main crack, resulting in a significant increase in load before a subsequent drop.

The first part of the loading curve for the three different configurations exhibited a linear elastic trend. N_2_/H_2_ specimens showed higher crack propagation resistance than N_2_ and NT specimens, but N_2_ specimens displayed less instability during crack propagation. This trend validates the observations derived from the surface characterization, particularly regarding the oxidative surface action achieved and its uniformity with the N_2_-based treatment compared to the strongly reducing action of N_2_/H_2_. The load peaks recorded are higher in the latter case due to the greater functionalization density. XPS analysis indeed revealed a higher presence of functional groups (i.e., carboxylic, carbonylic, etc.) that can react effectively with the resin epoxy groups, allowing for greater energy absorption before crack propagation. Conversely, in the N_2_ case, the functionalization density is lower but more homogeneous, enabling better recovery of the fiber-matrix adhesive integrity, thus showing a more continuous pattern.

**Figure 9:**
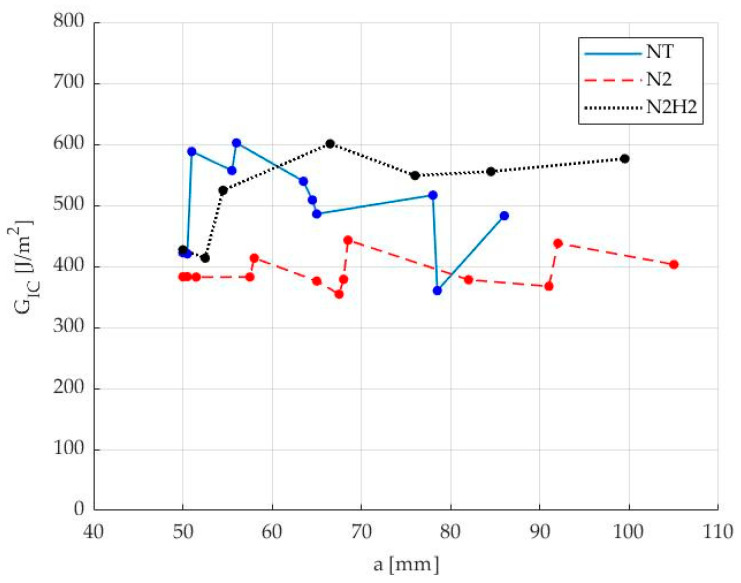
*R* curves for untreated NT sample (blue), N_2_ (red) and N_2_/H_2_ (black) treated sample, as *G_IC_* vs. *a* crack tip position.

CC parameters were derived from load–displacement data to calculate fracture toughness values. The crack propagation resistance curves, R curves, were obtained as a function of the corresponding crack lengths, an example of which is reported in Figure 9 showing the first sample of each batch of specimens.

As a further confirmation of the stick-slip phenomenon, the NT case exhibits a more irregular and distinct “zig-zag” pattern of the *G_IC_* as a function of the position of crack “a”.

N_2_/H_2_ specimens exhibited a 14.3% increase in maximum peak force and a 3.3% increase in first propagation *G_IC_* value, while N_2_ specimens revealed a 16.7% decrease in *P_max_* and a 17.3% decrease in first *G_IC_* but a more stable trend. Table 6 summarizes the main results, including the average values of *P_max_* and *G_IC_* at first propagation *a*_0_ and subsequent ones a_i_, and their standard deviation in brackets.

#### 3.2.2. Fracture Toughness *G_IIC_*–End-Notched Flexure Test

**Figure 10:**
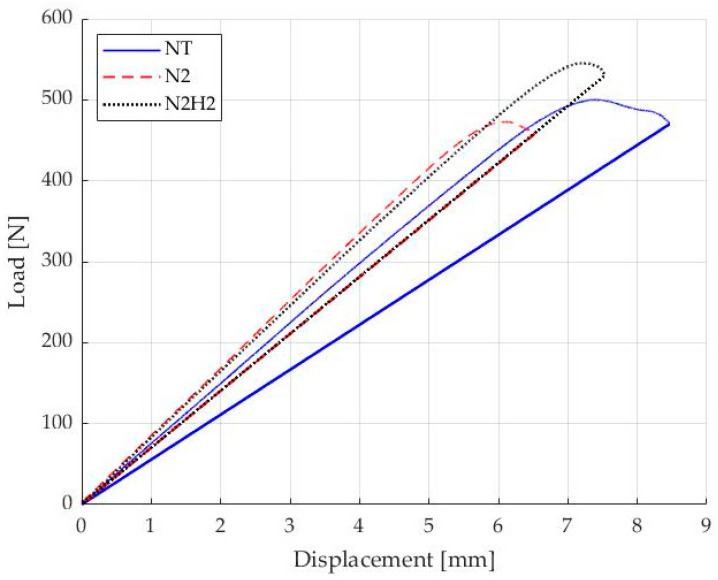
ENF-PC test–Load vs. displacement for untreated NT sample (blue), N_2_ (red) and N_2_/H_2_ (black) treated sample.

Load–displacement curves of specimens fabricated from untreated and plasma-treated fabrics are shown in Figure 10, displaying the PC fracture test phase. The curve shows the loading condition until the limit load is reached as described by the standard: the curve representing the N_2_ and N_2_/H_2_ cases has a similar slope, different from that for the NT case. CC was used to calculate candidate toughness *G_Q_* and then for *G_IIC_* according to the standards. 

The %*G_Q_*, defined in Section 2.2.6, evaluation results are shown in Table 7, while the results of ENF tests are listed in Table 8, along with standard deviation in brackets. All the computed %*GQ* were found to be within the acceptable range, so candidate toughness is coincident with *G_IIC_*, as suggested in ASTM standard procedure.

The N_2_/H_2_ specimens, compared with the NT ones, returned an increase of 16.1% for the PC *G_IIC_*, even if the two samples, N_2_/H_2#1_ and N_2_/H_2#2_, returned slight differences as confirmed by the STD.

On the contrary, the N_2_ specimens did not offer improvements compared to the NT specimens, confirming the results obtained for the Mode I delamination test by DCB test. The decrease in *G_IIC_* values compared to NT was 7.8% for PC *G_IIC_*.

#### 3.2.3. Flexural Test

**Figure 11:**
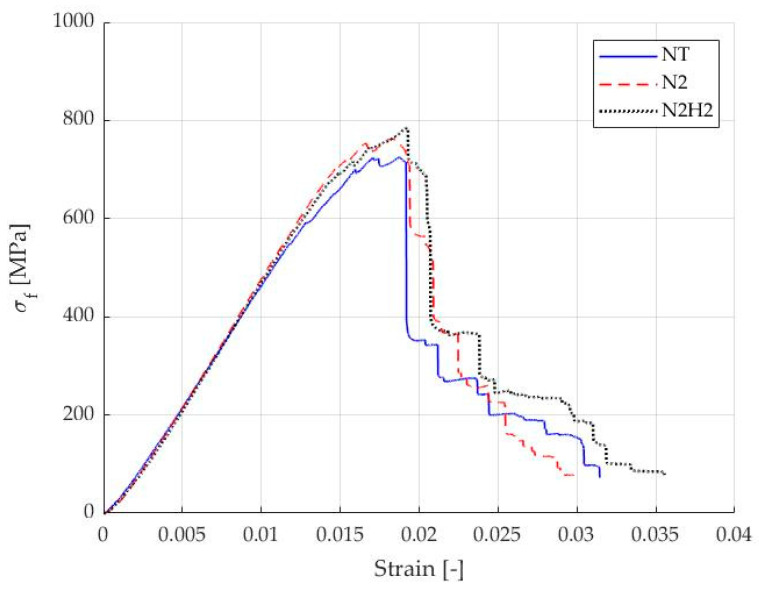
Flexural test–Stress vs. strain for untreated NT sample (blue), N_2_ (red) and N_2_/H_2_ (black) treated sample.

In this section, the results of the flexural tests conducted on the three different specimen configurations are presented. Stress–strain curves were obtained for each specimen, illustrating the mechanical response under bending conditions, as shown in Figure 11.

The stress–strain curves present similar slopes in the initial part of the curves, while the average maximum stress is higher for the specimens prepared with the plasma-treated carbon fibers. ANOVA statistical analysis on stress-related results was conducted, and the differences in maximum stress were found to be significant. The corresponding flexural stress and bending modulus values were calculated and are summarized in Table 9, along with their respective standard deviations shown in brackets.

Flexural stress at max load and modulus increased with N_2_ and N_2_/H_2_ plasma treatment compared to the tested samples fabricated with the as-received carbon fabric and epoxy resin as a matrix. In the N_2_ case, *σ_f_* increased by 3.5% while *E_b_* increased by 4.6%. On the other hand, in N_2_/H_2_ case, the *σ_f_* increased by 4.9%, while *E_b_* enhanced by 3.1%.

This implies that the APPJ treatment can activate CF fabric surfaces and slightly enhance the CF/epoxy interfacial strength.

## 4. Conclusions

In this work, Atmospheric Pressure Plasma Jet (APPJ) surface treatment of carbon-fiber (CF) reinforcement fabrics was carried out to enhance fiber-matrix adhesion characteristics.

Preliminary analysis has been conducted on the plasma source with Optical Emission Spectroscopy (OES) assessing the potential of the technology in terms of grafting chemical elements onto the surface that could enhance the chemical-physical interaction with the adopted Bio-resin (31% of bio content). In accordance with this, the best setup of plasma process parameters was chosen in terms of power and gas flow rate for the surface treatment of reinforcement fabrics, before the vacuum fabrication process. In tandem, surface characterizations were carried out by micro-Raman, Fourier Transform Infrared-Attenuated Total Reflection (FTIR-ATR) and X-ray Photoelectron Spectroscopy (XPS) on fabric samples. Subsequently, samples were obtained from the final laminates for mechanical characterization tests.

The plasma treatment has successfully improved the interlaminar fracture toughness in Mode I and Mode II and in N_2_/H_2_ gas choice condition, as confirmation of chemical-physical surface characterization made by FTIR-ATR, micro-Raman and XPS analysis.

A 14.3% increase in maximum peak force and a 3.3% increase in first propagation *G_IC_* value was registered with the Double Cantilever Beam (DCB) test while a 16.1% increase in precracked (PC) *G_IIC_* has been reported with ENF test. 

The action of the N_2_/H_2_-based plasma appears to have not only removed traces of the surface binder present in the tissue as received by the handling function but also acted at the structural level in reorganizing carbon-based chemical bonds and modifying the interactions of the carbon crystalline planes.

The flexural test properties were also enhanced compared to the initial condition without surface treatment of the reinforcement fabrics. This implies that the APPJ treatment was successful in activating and functionalizing the surface of the CF fabrics and increasing the interfacial strength of the CF/epoxy composite.

Conversely, the N_2_ case did not show substantial benefits in terms of improvements in the mechanical strength of the final composite other than a slight increase in flexural performance. 

In contrast to what was shown for the N_2_/H_2_ case, the action of the plasma on the treated fabric seems to have been predominantly oxidizing by promoting ablation of the surface binder.

Crack propagation appears to be more uniform due to a higher consistency in the surface treatment, unlike the N_2_/H_2_ case which instead exhibited a higher functionalization density, and thus a greater presence of functional groups that chemically bond more effectively to the resin.

APPJ technology has demonstrated its potential, achieving promising results, and it is not limited to pilot/prototype applications, as the machine used had a pre-industrial setup that was suitable for larger-scale utilization. Nevertheless, some application limitations have been considered, such as the maximum encumbrance such that sheets and reinforcing fabrics no larger than 35 × 35 cm could be processed in plane, an aspect resolved on plants acting on continuous production lines with adequate dimensions.

The results obtained only partially confirmed the potential of the adopted technological setup, stressing the need to adopt a more rigorous and mathematical method to find optimized plasma parameters able to maximize mechanical properties and increase energy efficiency. The authors are already working on the definition of a Design of Experiment (DoE) that can predict the treatment outcome by exploring a wide range of plasma parameters to be applied. This would go towards a decrease in prototype testing to ascertain the efficiency of the treatment thanks to the data predicted by the model, calibrated to provide a result with a high degree of reliability.

## Figures and Tables

**Table 1:** IB2 resin properties [16].

	IB2
Tensile strength [MPa]	65.0
Flexural strength [MPa]	107.0
Flexural modulus [MPa]	2.8
Elongation at Break [%]	5.3

**Table 2:** Process parameters for N_2_ and N_2_/H_2_ plasma treatments on CF fabrics.

Process Gas	Power [W]	Distance Jet-Sample [mm]	Jet Speed [mm/s]	Gas Flow Rate [L/min]	Reference Voltage [%]	Plasma Voltage [V]	Plasma Intensity/Frequency [kHz]
N_2_	660	12	25	31	100	334	25
N_2_/H_2_	810	16	25	31	100	334	25

**Table 3:** Peak shifts of band G and D1 after N_2_ and N_2_/H_2_ plasma treatments.

Peak Shift	D1 [cm^−1^]	G [cm^−1^]	D1–Shift	G–Shift
NT	1376.9	1589.9	-	-
N_2_	1371.7	1592.0	-	-
NT → N_2_	-	-	−5.2	+2.1
NT	1376.9	1589.9		
N_2_/H_2_	1379.3	1586.1		
NT → N_2_/H_2_			+2.4	−3.8

**Table 4:** XPS C1s, O1s atomic percentages, O/C ratios for NT samples, N_2_ and N_2_/H_2_ treated samples.

	C1s [%]	O1s [%]	O/C
NT	81.1	18.9	0.23
N_2_	74.1	25.9	0.35
N_2_/H_2_	87.8	12.2	0.14

**Table 5:** XPS deconvolutions results of C1s and O1s core lines: different components with relative bindings energies and percentages.

	NT	N_2_	N_2_/H_2_
C1s	C-C (284.56 eV, 51.13%)	C-C (284.70 eV, 70.04%)	C-C (284.67 eV, 50.83%)
	C-O-C/C-OH (286.20 eV, 48.87%)	C-O-C/C-OH (286.36 eV, 17.29%)	C-O-C/C-OH (285.72 eV, 27.69%)
		C=O (288.23 eV, 7.55%)	C=O (287.21 eV, 6.64%)
		COC=O/HOC=O (289.77 eV, 4.32%)	COC=O/HOC=O (288.63 eV, 6.83%)
		π-π (292.34 eV, 0.79%)	π-π (291.86 eV, 8.01%)
O1s	C-O (532.96 eV, 100.00%)	C-O (532.48 eV, 55.79%)	C-O (532.73 eV, 67.15%)
		C=O (531.38 eV, 32.24%)	C=O (530.85 eV, 19.58%)
		COOH (533.74 eV, 11.97%)	COOH (534.92 eV, 13.28%)

**Table 6:** Main parameters average values as output of DCB test.

Average	*P_max_* [N]	*G_IC_ a*_0_ [J/m^2^]	*G_IC_ a_i_* [J/m^2^]
NT	40.1 (2.0)	430.1 (28.8)	546.0 (41.6)
N_2_	33.3 (1.7)	355.6 (63.2)	379.7 (42.9)
N_2_/H_2_	45.8 (1.1)	444.1 (24.1)	539.7 (26.6)

**Table 7:** The %*G_Q_* for sample NT, N_2_ and N_2_/H_2_ batches.

	%*G_Q_* NT	%*G_Q_* N_2_	%*G_Q_* N_2_/H_2_
NPC (*a*_0_ = 20 mm)	17.8% (2.0)	18.8% (2.9)	26.0% (3.9)
NPC (*a*_0_ = 40 mm)	22.6% (3.9)	23.3% (7.6)	27.5% (8.6)
PC (*a*_0_ = 20 mm)	16.7% (0.7)	17.7% (3.3)	25.4% (3.7)
PC (*a*_0_ = 40 mm)	18.5% (3.5)	25.0% (6.4)	29.0% (1.6)

**Table 8:** Average *P_max_* and *G_IIC_* of first crack propagation for the tested batches.

Sample	Test	*P_max_* [N]	*G_IIC_ a*_0_ [J/m^2^]
NT	PC	504.5 (9.2)	1420.5 (36.0)
N_2_	PC	467.8 (34.9)	1309.7 (75.3)
N_2_/H_2_	PC	505.8 (56.0)	1649.4 (133.6)

**Table 9:** Main parameters average values as output of Flexural test.

Average	*σ_fmax_* [MPa]	*E_b_* [MPa]
NT	719.0 (9.15)	45.2 (2.2)
N_2_	744.6 (26.5)	47.3 (2.8)
N_2_/H_2_	754.3 (42.4)	46.6 (2.9)

## Data Availability

Data are contained within the article.
